# Diversity and temporal distribution of *Fusarium oxysporum* f. sp. *vasinfectum* races and genotypes as influenced by *Gossypium* cultivar

**DOI:** 10.3389/ffunb.2022.1022761

**Published:** 2022-10-20

**Authors:** David R. Dyer, Molli Newman, Kathy S. Lawrence

**Affiliations:** ^1^ Department of Entomology and Plant Pathology, Auburn University, Auburn, AL, United States; ^2^ Biological and Environmental Sciences Department, Troy University, Troy, AL, United States

**Keywords:** cotton, *Fusarium oxysporum* f. sp. *vasinfectum*, FOV, genotype, *Gossypium*, *Meloidogyne incognita*, root-knot nematode

## Abstract

This study assess the population diversity and temporal variability of caused by *Fusarium oxysporum* f. sp. *vasinfectum* (FOV) races/genotypes infecting cotton cultivars with either FOV or *Meloidogyne incognita* resistance. All plants sampled demonstrated typical symptoms of FOV including wilting, chlorosis and necrosis of the leaves, and discoloration of the vascular tissue in the stem. A diverse population of FOV was characterized. Eight races/genotypes of FOV were collected throughout the three site years. FOV race 1 was the most predominant in all tests (AUDPC=101.1); statistically higher numbers of isolates from LA-108 (AUDPC=59.9), race 8 (AUDPC=47.5), and race 2 (AUDPC=38.6) were also found compared to other races and genotypes collected. FOV race 1, race 2, race 8, and 108 were the most virulent races identified. The genotypes MDS-12, LA-110, and LA-127/140 were found in all tests but at a low incidence, and LA-112 was only found in trace amounts. MDS-12, LA-110, LA-112, and LA-127/140 produced less disease pressure. FOV race 4 which is highly virulent and present in California and Texas was not found in Alabama. A positive correlation was observed between the accumulation of growing degree days and FOV race 1, race 2, race 8, LA-108, and LA-110. Later symptom expression influenced by seasonal heat partially mitigates damage allowing cotton to produce bolls though they may be reduced in number and lint quality. Plant resistance to the FOV as expressed in these cultivars appears to provide better protection than *M. incognita* resistance. PhytoGen 72, which is resistant to FOV races/genotypes had low levels of FOV infection even though it sustained a high level of *M. incognita* root population density. The *M. incognita* resistant cultivars Deltapine 1558NR B2RF and PhytoGen 480 W3FE supported a lower nematode population density, however, FOV disease incidence was not reduced. FOV races/genotypes did not vary significantly between the nematode resistant and nematode susceptible cultivars.

## Introduction

*Fusarium* wilt is caused by the seed and soilborne fungal pathogen *Fusarium oxysporum* that engenders disease in many crops worldwide. This species has more than 140 *forma speciales* capable of causing disease in plants belonging to more than 45 families (Edel-Hermann and Lecomte, 2019). *Fusarium oxysporum* f. sp. *vasinfectum* (Atk.) W.C. Snyder & H.N. Hansen (FOV) is a cotton pathogen that was first reported in 1892 from samples collected in Alabama and Arkansas (Atkinson, 1892). Fusarium wilt affects cotton in every major cotton-growing region worldwide (Davis et al., 2006). Atkinson noted that incidence and severity of *Fusarium* wilt, which was known as Frenching at the time, increased when plant also had root galling symptoms characteristic of the root-knot nematode, (RKN) *Meloidogyne incognita* (Kofoid & White) Chitwood. The disease complex formed between these two pathogens can result in severe foliar wilt symptoms and crop yield losses. The interactions of the nematode and the *Fusaria* are poorly understood and are a focus of ongoing research. The presence of *M. incognita* in this disease complex is believed necessary for several of the *Fusaria* known races/genotypes of FOV to cause significant damage, but the association is not required for all FOV (Liu et al., 2011; Bell et al., 2017). Specifically, *M. incognita* is believed to be required for FOV infection for all FOV races and genotypes except for the FOV race 4.

The National Cotton Council of America monitors yearly cotton disease losses across the United States, and *Fusarium* wilt has been a disease problem in all cotton producing states with a long history of economic losses. Estimates of the FOV yield losses for 2021 amounted to 15 million dollars (https://loss.cropprotectionnetwork.org) (Crop Protection Network, 2022). Losses due to this disease have been monitored over the last 56 years (1965-2021) with an average annual yield loss of 62,391 bales valued at 29.9 million dollars.

FOV is characterized by diverse races (Armstrong and Armstrong, 1958), vegetative compatibility groups (VCGs) (Fernandez et al., 1994; Davis et al., 1996; Bell et al., 2017), and several genotypes of the FOV pathogen (Holmes et al., 2009). Races 1, 2, 3, 4, 6, and 8, as well as genotypes LA-108, LA-110, LA-127/140, and MDS-12 are documented from the United States.

Smith (2015) reported that a single cotton field could contain multiple races/genotypes of the pathogen. However, little is known about how these races of FOV interact with one another within a field. Some races, such as FOV race 4, are thought to infect early in the first few weeks of the season, while others are known to infect cotton throughout the season (Hutmacher et al., 2011). This study was initiated at the National Cotton *Fusarium* Wilt Evaluation Field, in Tallassee, Alabama to 1) assess the population diversity of FOV races/genotypes infecting cotton cultivars selected for genetic resistance to FOV or *M. incognita* and to 2) determine the in-season temporal variability of FOV races/genotypes that might be isolated from these selected cotton cultivars.

## Materials and methods

### Field location

Testing was conducted at the Plant Breeding Unit of Auburn University’s E. V. Smith Research Center in Tallassee, AL in the National Cotton *Fusarium* Wilt Evaluation Field (latitude 32° 29’20.68” N longitude 85° 52’59.04” W). This field is dedicated to evaluate new cotton genotypes and breeding lines for their resistance or susceptibility to the *Fusarium* wilt-*M. incognita* disease complex for more than 60 years (Kathryn Glass, personal communication). The trial field is a Kalmia loamy sand soil type consisting of 80% sand, 10% silt, and 10% clay with 1% organic matter and a pH of 5.3. Past testing has revealed this field has a diversity of FOV races/genotypes (Smith, 2015), and it is known to contain an established population of *M. incognita* race 3 (Groover et al., 2020).

### Layout of experiments

Three cotton tests were planted on May 17, 2018 (one trial) and April 24, 2019 (two trials). All trials were planted in a Latin Square design with ten replications using a John Deere MaxEmerge (John Deere; Moline, IL) planter with Almaco cone planters (Almaco; Nevada, IA) at a seeding rate of 13.1 seeds/meter of row. Of the two tests planted in 2019, one was planted into the same area used in the 2018 testing, and one was planted in a part of the field that was previously planted in *Glycine max*. Each test plot consisted of one row that was 7.6 meters long with a 0.9-meter row spacing and a 1.8-meter alley between each replication.

### Cotton cultivars tested

Each plot was planted with one of eight cotton cultivars (**Table 1**) chosen for this test based on their resistance or susceptibility traits for either FOV or *M. incognita*. The cotton species *Gossypium hirsutum* and *G. barbadense* were included due to documented differences in their sensitivity to some races/genotypes of FOV (Kim et al., 2005). Upland cotton, *G. hirsutum*, cultivars ‘Rowden’ and ‘M-315’ were the susceptible and resistance checks, respectively, and were included in the test twice to help standardize the disease incidence across the field tests. The ‘Rowden’ (USDA-ARS; Washington, DC) cultivar is susceptible to both FOV and *M. incognita* infection (Doan and Davis, 2014). ‘M-315’ is a cultivar resistant to *M. incognita* infection (Shepherd et al., 1996). ‘Phytogen 480 W3FE’ (Corteva Agriscience; Wilmington, DE) and ‘Deltapine 1558NR B2RF’ (Bayer Crop Science; Leverkusen, Germany) are both resistant to *M. incognita* with two genes for resistance (Faske et al., 2018). ‘Stoneville 4946 GLB2’ (BASF corporation; Ludwigshafen, Germany) is a moderately resistant cultivar with one gene for resistance to *M. incognita*. One Acala cultivar, ‘Phytogen 72’ (PhytoGen Cottonseed, Indianapolis, Indiana, United States), is resistant to FOV, except for FOV race 4, and susceptible to *M. incognita* (Hutmacher et al., 2013). Two Pima, *G. barbadense*, cultivars were also included in the test. ‘PhytoGen 800’ (PhytoGen Cottonseed, Indianapolis, Indiana, United States), is resistant to FOV race 4 but susceptible to other races/genotypes of FOV, and susceptible to *M. incognita* infection (Mahill and Pellow, 2005). ‘Pima S-7’ (USDA-ARS; Washington, DC) is susceptible to both FOV and *M. incognita* (Turcotte et al., 1992).

**Table 1:** List of cotton cultivars and their reported resistance traits to either *Fusarium oxysporum* f. sp. *vasinfectum* (FOV) or *Meloidogyne incognita* the root-knot nematode (RKN).

Cotton Cultivar	Reported Resistance Traits	
Upland Cotton – *Gossypium hirsutum*		Source
Rowden	FOV and RKN susceptible	USDA-ARS; Washington, DC, United States
M-315	RKN resistant	USDA-ARS; Washington, DC, United States
PhytoGen 480 W3FE	RKN resistant	Corteva Agriscience; Wilmington, DE
DeltaPine 1558NR B2RF	FOV susceptible and RKN resistant	Bayer Crop Science; Leverkusen, Germany
Stoneville 4946 GLB2	RKN moderate resistance	BASF corporation; Ludwigshafen, Germany)
**Acala Cotton - *Gossypium hirsutum***		
PhytoGen 72	FOV resistant, RKN susceptible	PhytoGen Cottonseed, Indianapolis, Indiana, United States
**Pima Cotton – *Gossypium barbadense***		
PhytoGen 800	FOV race 4 resistant, RKN susceptible	PhytoGen Cottonseed, Indianapolis, Indiana, United States
Pima S-7	FOV and RKN susceptible	USDA-ARS; Washington, DC, United States

### FOV sample collection

**Figure 1:**
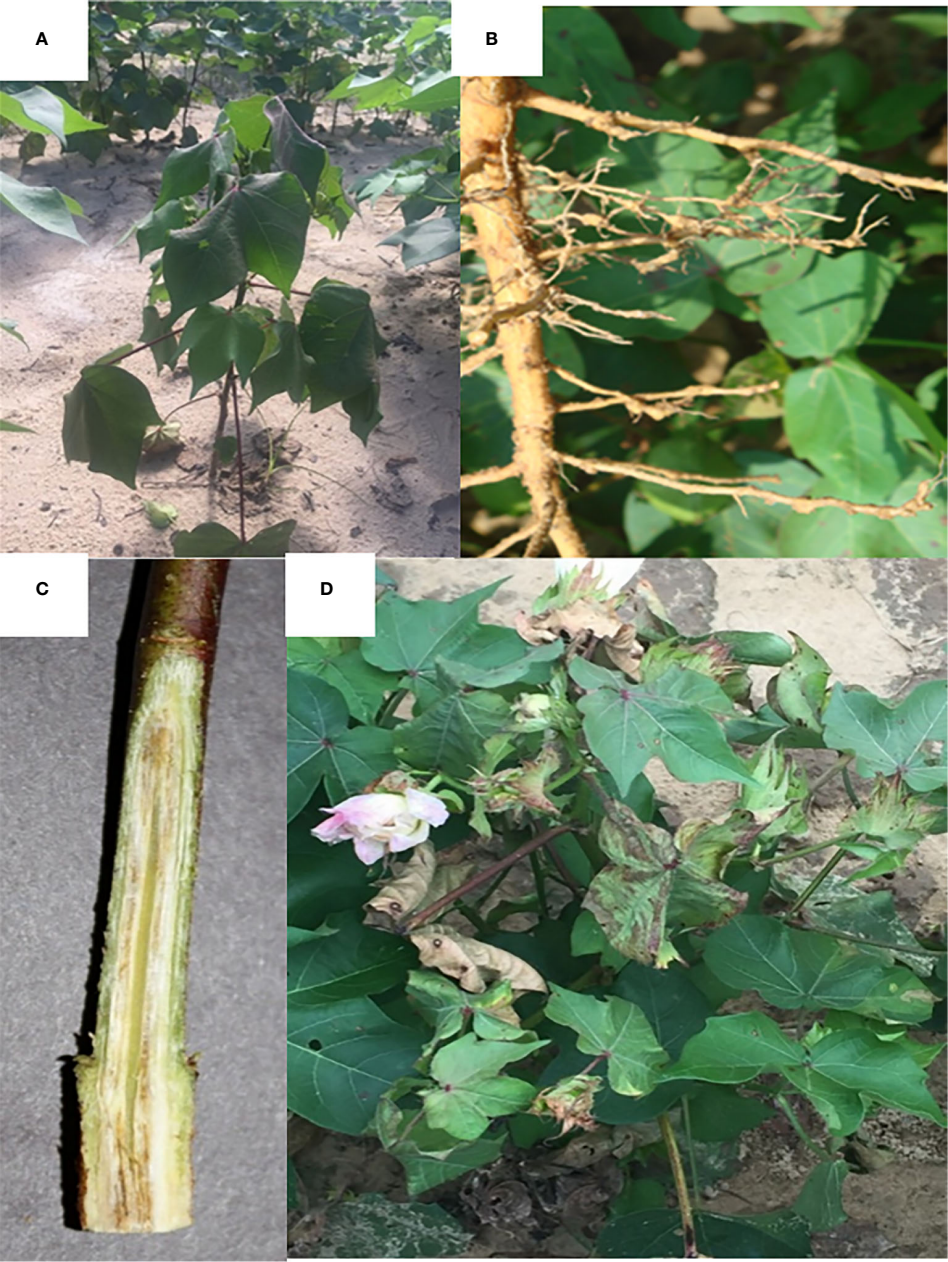
Signs and symptoms of *Fusarium* wilt on *Gossypium hirsutum*. **(A)** Wilting of cotton plant caused by *Fusarium* wilt infection. **(B)** *Meloidogyne incognita* galls on cotton roots. **(C)** Discoloration of the vascular tissue of a cotton stem. **(D)** Leaf necrosis and wilting caused by *Fusarium* wilt.

Sample collection of plants showing foliar symptoms (**Figure 1**) commonly associated with *Fusarium* wilt (wilting and chlorosis or necrosis of leaves) began two weeks after cotton planting. Sampling continued weekly for the first six weeks. After this time point, samples were taken every other week until the cotton was defoliated before harvest. Sampling in 2018 ranged from June 4 to September 10, and 2019 sampling ranged from May 8 until September 3. In all analyses, sampling dates are given growing degree day (DD60) values, as an alternative to calendar date, to account for both the time of the season and cotton growth (DeVay et al., 1997). DD60 for cotton are determined by averaging the daily high and low temperature and then subtracting 60 which is the base temperature for cotton. The value is the DD60 per day which is summed over the season. At each sampling date, any cotton plants from the test plots that exhibited foliar symptoms of *Fusarium* wilt were removed from the soil using a shovel and transported to the lab for isolation.

### FOV isolation

Fungal isolations were accomplished by splitting the lower stem and upper taproot of the plants using a scalpel. Three small sections of the vascular tissue were removed from each plant. Each section was surface sterilized in 95% ethanol for 30 seconds and a 0.625% NaOCl solution for 1 minute and placed onto a Petri dish containing half-strength acidified potato dextrose agar (APDA). The Petri dishes were incubated at room temperature for three to five days. X

### FOV morphological identification

Cultures morphologically identified as *Fusarium* were transferred to new half-strength APDA plates. *Fusarium* sporulation starts quickly in the aerial mycelium; microconidia arise from simple phialides born on short conidiophores. Microconidia are one celled, cylindrical to ellipsoid, and 5-20 x 2.2-3.5 µm in size. Macroconidia are falcate in shape, three to five celled, 27-48 x 2.5-4.5 µm in size and born on conidiophores or in sporodochium (Nelson et al., 1983). Cultures are stored in the Auburn University Microbial Collection and can be accessed through https://www.auburn.edu/iac.

### FOV molecular identification

Race identification of each FOV isolate collected throughout the growing season was obtained by sequencing a portion of three genes and comparing these to sequences from specific reference isolates. To extract genomic DNA the strains were transferred to a new half-strength APDA plate containing a sterile cellophane sheet (Bennett et al., 2013). After 5-10 days incubation, the mycelium was harvested from each culture by scraping the surface of the cellophane sheet with a sterile scalpel. DNA was extracted from this mycelium using a Quick-DNA Fungal/Bacterial Miniprep Kit (Zymo Research; Irvine, CA) following the manufacturer’s protocol. Samples of fungal DNA were stored at -20°C until further use.

Portions of the translational elongation factor (*EF-1*α), βeta-tubulin (*BT*), and phosphate permease (*PHO*) genes were sequenced for identification of the isolates. PCR amplifications were conducted in 0.2 mL PCR tubes containing 12.5 µL of JumpStart™ REDTaq^®^ ReadyMix™ reaction mix (Sigma-Aldrich; St. Louis, MO), 0.5 µL of each primer (10 mM), 1.5 µL of DNA template, and 10 µL of nuclease-free water. Primers and amplification conditions used are listed in **Table 2**. PCR products were sent to Eurofins Genomics (Louisville, KY) for purification and sequencing. Primers used for sequencing were the same as were used for amplification. Because *EF-1*α, *BT*, and *PHO* are not adequate to differentiate between FOV race 4 and MDS-12 (race 4-like) isolates (Scott, 2012; Bennett et al., 2013).

**Table 2:** Primers and thermocycler settings used for race identification of FOV isolates.

Primer name	Primer sequence	Reference	Thermocycler settings
**Translational elongation factor (EF-1α)**			
EF-1	ATGGGTAAGGAAGACAAGAC	(O’Donnell et al., 1998)	94°C for 2 min followed by 40 cycles of 95°C for 30 sec, 55°C for 30 sec, and 72°C for 1 min with a final extension of 72°C for 5 min
EF-2	GGAAGTACCAGTGATCATGTT		
**Beta-tubulin (BT)**			
BT 3	CGTCTAGAGGTACCCATACCGGCA	(Tooley et al., 2001)	94°C for 2 min followed by 35 cycles of 94°C for 30 sec, 52°C for 30 sec, and 72°C for 1.5 min with a final extension of 72°C for 10 min
BT 5	GCTCTAGACTGCTTTCTGGCAGACC		
**Phosphate permease (PHO)**			
PHO 1	ATCTTCTGGCGTGTTATCATG	(O’Donnell et al., 2000)	97°C for 1 min followed by 35 cycles of 96°C for 30 sec, 50°C for 1 min, and 72°C for 1 min with a final extension of 72°C for 10 min
PHO 6	GATGTGGTTGTAAGCAAAGCCC		
**Race 4 specific primers**			
R4F	GCTCCGTGTCWGAGCTTCTT	(Yang et al., 2006)	94°C for 3 min followed by 10 cycles of 94°C for 30 sec, 59°C for 30 sec, and 72°C for 30 sec, 25 cycles 90°C for 30 sec, 59°C for 30 sec, and 72°C for 15 sec with a final extension of 72°C for 1 min
R4R	TGCTCATCGTGGAGCATAAC		
**Multiplex PCR for detection of TƒO1, MULE/TƒO1, and MITE/TƒO1 insertions in the PHO gene**			
FovP-f	GGCCGATATTGTCGGTCGTA	(Ortiz et al., 2017 and Bell et al., 2019)	94°C for 2 min followed by 35 cycles of 94°C for 30 sec, 58°C for 30 sec, and 72°C for 40 sec with a final extension of 72°C for 5 min
FovM-R	CCGCCATATCCACTGAACA		
FovT-R	ATCTGTCTTTCGTCGGCAAT		
FovP-R	CTCCAGTGCAGTGCTTGGTA		
**Intergenic spacer regions (IGS)**			
CNS1	GAGACAAGCATATGACTAC	(O’Donnell et al., 2009)	94°C for 90 sec followed by 40 cycles of 94°C for 30 sec, 58°C for 90 sec, and 68°C for 3 min with a final extension of 68°C for 5 min
NL11	CTGAACGCCTCTAAGTCAG		
iNL11	AGGCTTCGGCTTAGCGTCTTAG		Used for sequencing only
NLa	TCTAGGGTAGGCKRGTTTGTC		
CNSa	TCTCATRTACCCTCCGAGACC		
iCNS1	TTTCGCAGTGAGGTCGGCAG		

All isolate identified as Race 4 and MSD-12 were screened using a three step approach. First, a multiplex PCR analysis using primers FovP-F, FovM-R, FovT-R, and FovP-R (**Table 2**) was conducted to look for insertions in the PHO gene of the isolates. Some isolates of FOV race 4 have been found to have a Tƒo1 transposon (Tƒo1), a mutator-like transposon element (MULE), or a miniature inverted-repeat transposable element (MITE) insertion in the PHO gene. After PCR amplification, samples were run through a 1.5% agarose gel electrophoresis. Isolates without a gene insertion produce a DNA fragment of 396 base pairs (bp), while an isolate containing one of the insertions will range from 426-663 bp (Ortiz et al., 2017; Bell et al., 2019). Secondly, PCR was conducted on all isolates using the FOV race 4 specific primer set R4F and R4R following the protocol outlined by Yang et al. (2006). Finally, nearly full-length sequences of the IGS gene were obtained using the primers listed in **Table 2** and following the protocol outlined by O’Donnell et al. (2009). These sequences were then compared to the same reference isolates used for the *EF-1*α, *BT*, and *PHO* sequences. DNA sequences were deposited in Genbank using BankIt and can be found under the following accession numbers ON409993-ON411130.

DNA sequencing results were aligned using BioEdit Sequence Alignment Editor and were manually adjusted (Hall, 1999). Sequence alignments were compared to previously published reference sequences downloaded from GenBank. Phylogenetic analyses were conducted using the software MEGAX (Kumar et al., 2018). A phylogenetic tree was constructed using the Maximum Likelihood method based on the Tamura-Nei model (Tamura and Nei, 1993). Branching patterns were determined by a bootstrap method with 1000 replicates.

### Nematode sampling and extraction

Nematode samples were collected at the end of each cotton growing season just before the defoliation of the cotton crop (September 24, 2018, and September 14, 2019). Samples were collected using shovels to remove the roots of two arbitrarily selected plants from each one-row trial plot. *M. incognita* eggs were extracted from the cotton roots by a modified version of the methodology of Hussey and Barker (1973). The cotton roots were placed in a 0.625% NaOCl solution and agitated or shaken for four minutes at 1 g force or 130 rpm using a Barnstead Lab-Line Max Q 5000E class shaker (Conquer Scientific; San Diego, CA) to remove the eggs from the roots. *M. incognita* eggs were washed from the roots with tap water and collected on a 25-µm pore sieve. Eggs were then separated from the soil by modified sucrose (1.14 specific gravity) centrifugation at 240 g (1400 rpm) for 1 minute (Jenkins, 1964).

### Nematode assessment

After extraction, nematode eggs were enumerated under a Nikon TSX 100 inverted microscope (Nikon; Tokyo, Japan) at 40x magnification. Eggs per gram of cotton root (Eggs/g of root) were calculated by taking the ratio of total eggs extracted per the fresh root weight.

### Statistical analysis

Statistical analysis was conducted in R (R Core Team, 2018) using RStudio (RStudio Team, 2015). *Fusarium* wilt incidence was collected from cotton trials throughout the three site years of the test. These data are presented as a disease index (DI) value based on a scale of 0-4 where each whole number corresponds to a 25 percent disease incidence (Schandry, 2017). The DI was calculated using the equation $\text{DI}=\frac{F}{T}*4$ , where F is equal to the total number of plants from which FOV was isolated and T is equal to the total plants in the plot obtained from 14 DAP stand counts (Schandry, 2017). For each FOV race/genotype, and each cultivar included in the test, the area under the disease progress curve (AUDPC) was calculated based on the calculated DI and DD60’s. AUDPC’s were calculated using the Agricolae package (Mendiburu, 2020) in R, and graphs were created using the ggplot2 package (Wickham, 2016). Analysis of AUDPC was conducted to compare both races of FOV and cotton cultivar by a linear mixed effect model, including test as a random effect in both analyses. Nematode eggs/g of root data were analyzed by ANOVA and means were separated using Tukey test at the *P* ≤ 0.05 level. Eggs/g of root data were log-transformed to satisfy the ANOVA assumption of normally distributed residuals.

## Results

### FOV races and genotypes

**Figure 2:**
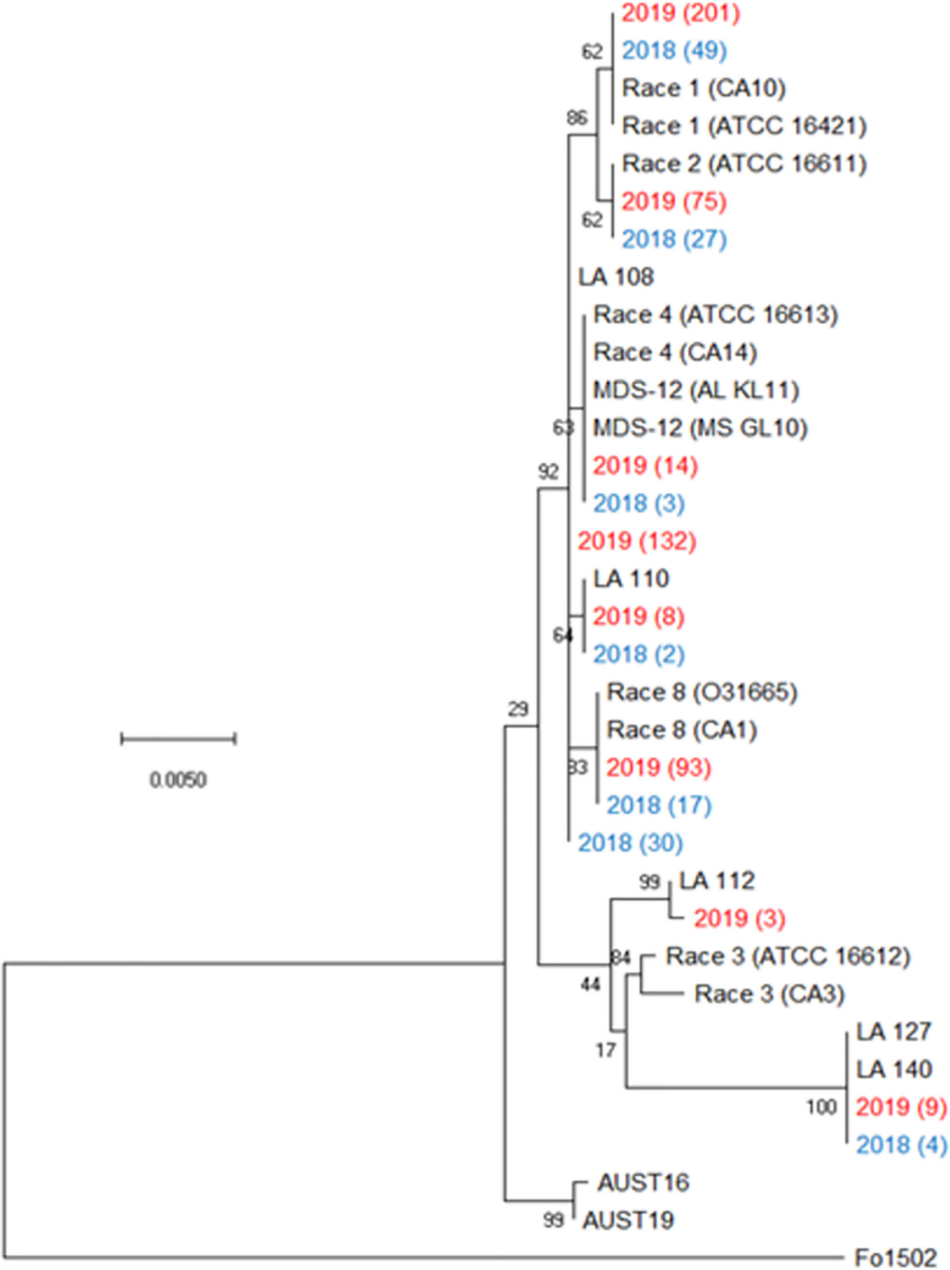
Condensed phylogenetic tree of FOV isolates collected during the 2018 and 2019 cotton seasons using a partial sequence analysis of the translation elongation factor, β-tubulin, and the phosphate permease genes. Tree was constructed in MEGAX using the maximum likelihood method based on the Tamura-Nei model (Tamura and Nei, 1993). The tree with the highest log likelihood (-2944.90) is shown. Bootstrap frequencies from 1,000 replications are noted next to every branch. Isolates with identical sequences collected during the study are represented by a single isolate in the tree and are labeled with the year of collection and the number of identical isolates that were found. For example, isolate 2019 (201) represents 201 identical isolates that were collected in 2019. Isolates collected during the 2018 season are shown in blue and isolates collected during 2019 are shown in red. References isolates (in black) used for comparison are labeled by the race followed by an isolate name, for example Race 1 (CA10) is a race 1 reference isolate identified as CA 10. A non-pathogenic *Fusarium oxysporum* (isolate 1502) was used as an outgroup to root the tree.

**Figure 3:**
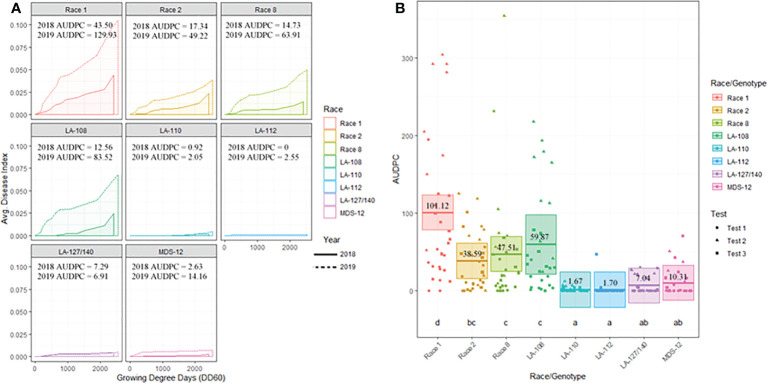
**(A)** Temporal disease progression curves for each race/genotype of FOV collected to show the distribution throughout the cotton seasons depicted as a solid line for 2018 testing and a dashed line for 2019 testing. Area under the disease progress curve (AUDPC) is shaded in a lighter color as indicated by the legend. **(B)** Box plots with means (indicated by thick horizontal lines) and 95% confidence intervals (shaded boxes) of the AUDPC values for each race/genotype of FOV as estimated by a linear mixed effect model shown as an average of all three test. Calculated AUDPC values for individual test plots are marked by symbols corresponding to the test from which they were collected. The mean AUDPC value is listed just above the mean line for each race/genotype of FOV and statistical significance is indicated by letters below the boxplots. Races/genotypes that share letters do not differ significantly.

All plants sampled demonstrated typical symptoms of FOV including wilting, chlorosis and necrosis of the leaves, and discoloration of the vascular tissue in the stem (**Figure 1**). From the plants sampled during the three site years, FOV isolates were successfully obtained from 661 plants. Of these isolates, 126 were obtained during the 2018 test, and 535 were recovered during 2019. From the 661 total isolates seven races/genotypes were identified during the 2018 test, and eight were collected during the 2019 test (**Figure 2**). **Figure 3A** displays the average AUDPC of each race/genotype in 2018 compared to the 2019 cotton season. Increased disease incidence was observed for each race/genotype during 2019 as compared to 2018. In this analysis, the curves are an average of all replications of the tests conducted in the respective year. These data represent ten replications in 2018 and twenty replications in 2019.

**Figure 3B** illustrates the statistical analysis of the AUDPC for each of the eight races found during these two years. Individual AUDPC values for each replication are marked with symbols that correspond to each test. The average AUDPC values and the 95% confidence intervals for each race/genotype, based on a linear mixed-effect model, are indicated with shaded boxes. FOV race 1 was the most damaging race with an average AUDPC of 101.1 and the most common field isolate in all tests making up 37.7% of the total FOV isolates (**Figure 3B**). However, high levels of damage were sustained from genotype LA-108, race 8, and race 2 with average AUDPC values of 59.8, 47.5, and 38.5, respectively. The genotypes LA-110 and LA-127/140 were found at low levels, 10 and 13 isolates respectively, during both years of testing. Three isolates of the genotype LA-112 were found in one of the 2019 tests (the location not used in 2018). This genotype was not found in 2018. In all three site years, a total of 16 isolates shared a 100% identity with FOV race 4 and MDS-12 when sequenced at the *EF-1*α, *BT*, and *PHO* gene regions. Sequencing at these three gene regions using the primers listed in **Table 2** does not allow distinguishing between the highly virulent FOV race 4 and the less virulent MDS-12 isolates.

**Figure 4:**
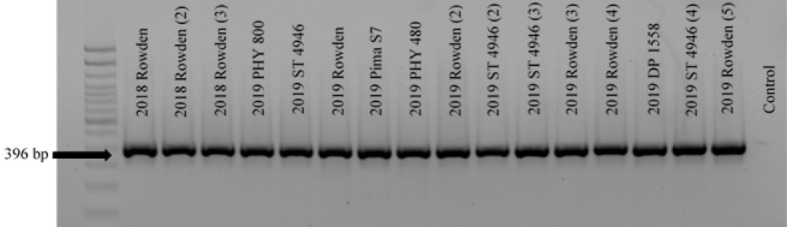
Gel electrophoresis image of a PCR analysis for the detection of Tƒo1, MITE/Tƒo1, MULE/Tƒo1 insertions into the PHO gene of FOV isolates which are commonly found in FOV race 4 isolates in the United States. Bands appear at 396 bp when no insertion is present, 583 bp when the Tƒo1 insertion is present, 426 bp when the MULE/Tƒo1 insertion is present, and 663 bp when the MITE/Tƒo insertion is present. Isolates shown are lane 1, 100 bp DNA ladder; lane 2-17, MDS-12 isolates of FOV; lane 18 water control. All isolates produced a band at 396 bp showing the lack of a Tƒo insertion into the PHO gene.

**Figure 5:**
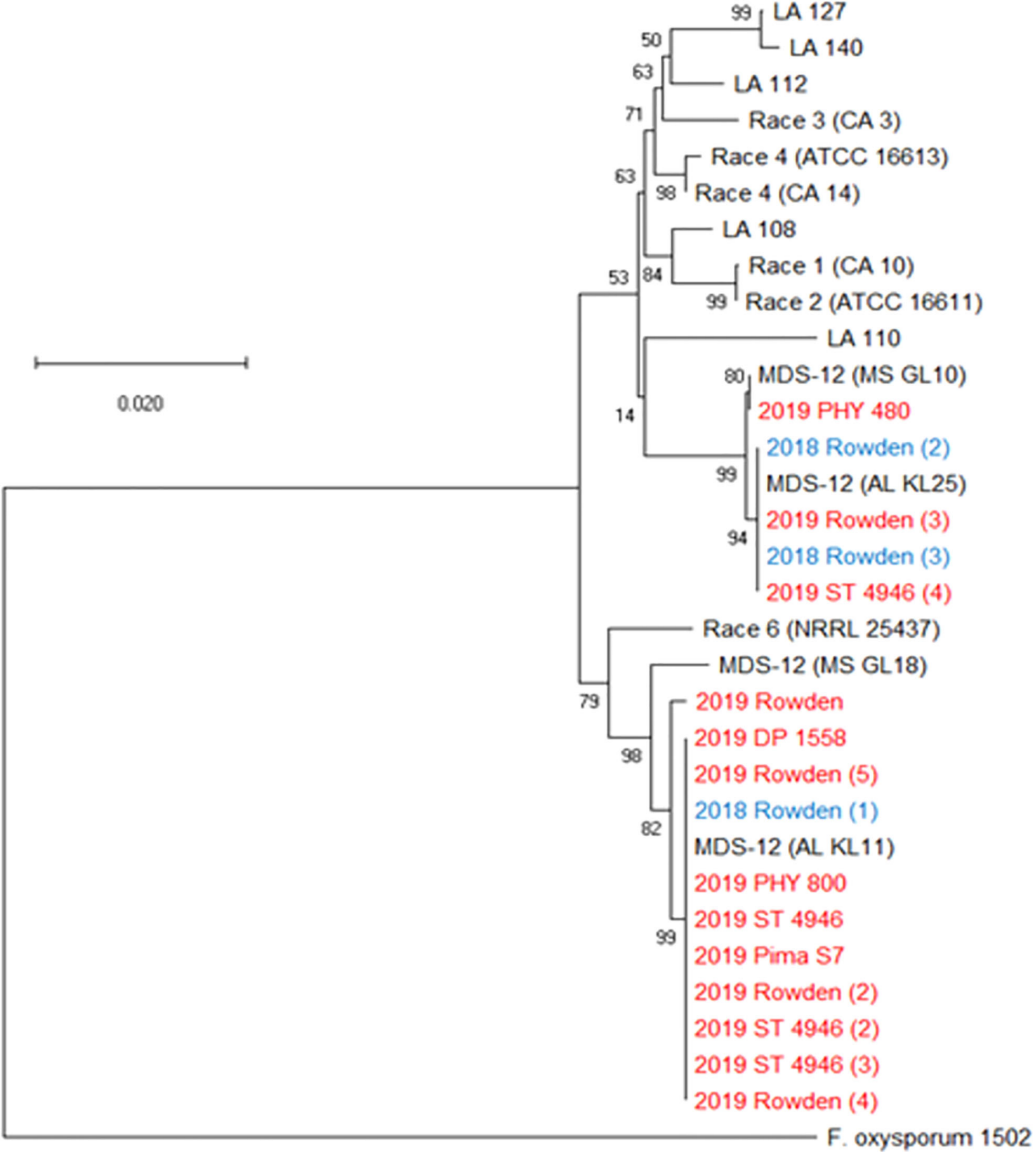
Phylogenetic tree of FOV isolates collected during the 2018 and 2019 cotton seasons using a partial sequence analysis of the translation elongation factor and nearly full-length sequences of the intergenic spacer region. This tree only shows isolates that were identified as FOV race 4 and MDS-12 when sequenced at the translation elongation factor, β-tubulin, and the phosphate permease gene regions, and thus required further sequencing for identification. Tree was constructed in MEGAX using the maximum likelihood method based on the Tamura-Nei model (Tamura and Nei, 1993). The tree with the highest log likelihood (-7043.88) is shown. Bootstrap frequencies from 1,000 replications are noted next to every branch. Isolates are labeled with the year that they were collected followed by the cultivar from which they were collected, in the case of isolates having the identical name, a number was added in parenthesis to separate isolates. For example, isolate 2018 Rowden (2) represents the second isolate collected from the Rowden cultivar in 2018. Isolates collected during the 2018 season are shown in blue and isolates collected during 2019 are shown in red. Reference isolates (in black) used for comparison are labeled by the race followed by an isolate name, for example Race 1 (CA10) is a race 1 reference isolate identified as CA 10. A non-pathogenic *Fusarium oxysporum* (isolate 1502) was used as an outgroup to root the tree.

Three further steps were taken to identify these isolates as either FOV race 4 or MDS-12. In a multiplex PCR analysis to look for Tƒo transposon insertions in the PHO gene, all of the isolates produced an amplicon of 396 bp (**Figure 4**) regardless of the year of collection or cotton variety from which they were isolated. This 396 bp fragment is equivalent to the DNA fragment size expected for MDS-12 and by some FOV race 4 isolates. In the PCR analysis using FOV race 4 specific primers, there was no amplification for any of the isolates in question. IGS sequences of these isolates shared 100% identity with previously identified MDS-12 isolates. A phylogenetic analysis of the combined *EF-1*α and IGS genes grouped the isolates into two separate clades with previously identified MDS-12 isolates AL KL 11, AL KL 25, MS GL 10, and MS GL 18 (**Figure 5**). FOV Race 4 isolates included in the analysis were contained in a separate clade (**Figure 5**), demonstrating that the 16 isolates in question did not match FOV race 4.

### Effect of cotton cultivar and *M. incognita*

**Figure 6:**
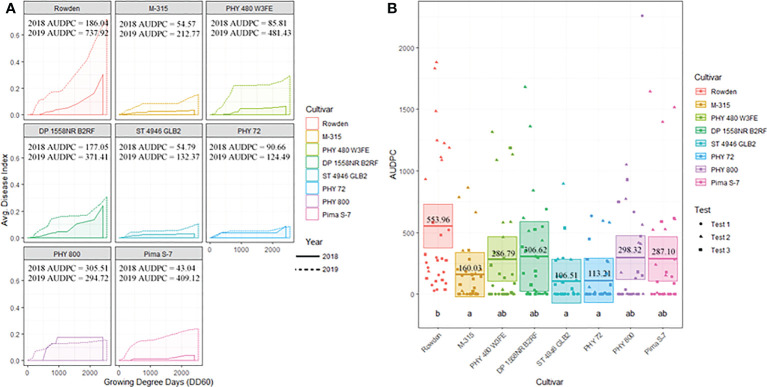
**(A)** Temporal disease progression curves for each cultivar included in the test to show the distribution throughout the cotton seasons demonstrated as a solid line for 2018 testing and a dashed line for 2019 testing. Area under the disease progress curve (AUDPC) is shaded in a lighter color as indicated by the legend. **(B)** Box plots means (indicated by thick horizontal lines) and 95% confidence intervals (shaded boxes) of the AUDPC values for each cotton cultivar as estimated by a linear mixed effect model shown as an average of all three test. Calculated AUDPC values for individual test plots are marked by symbols corresponding to the test from which they were collected. The mean AUDPC value is listed just above the mean line for each race/genotype of FOV and statistical significance is indicated by letters below the boxplots. Races/genotypes that share significance letters do not differ significantly.

Cotton cultivar played a significant role in the prevalence of FOV infection throughout the study. **Figure 6A** displays the average AUDPC values for each cotton cultivar included in the 2018 and 2019 seasons to demonstrate the variation of disease impact between the two years. In this analysis, the curves are an average of all test plots containing each cultivar in each year. During both years of testing Rowden, the susceptible check, sustained increased incidence of *Fusarium* wilt throughout the entirety of the cotton season. Other cultivars included in the test were affected by FOV at varying levels. All cultivars included in the test had a higher AUDPC value during the 2019 cotton season, except for PHY 800 (**Figure 6A**). This cultivar had a higher AUDPC rating for the 2018 season where generally less FOV infection was observed.

**Figure 6B** illustrates the statistical analysis of the AUDPC for each of the eight cotton cultivars included in the two years of testing. In this Figure, individual AUDPC values for each replication are marked with symbols that correspond to each test plot. The average AUDPC value and 95% confidence intervals for each cotton cultivar based on a linear mixed-effect model are indicated with shaded boxes. Statistically similar AUDPC ratings were observed for Rowden (554.0), Deltapine 1558NR B2RF (306.6), PhytoGen 480 W3FE (286.8), PhytoGen 800 (298.3), and Pima S-7 (287.1) cultivars. PhytoGen 800 and Pima S-7, two *G. barbadense* cultivars, supported the highest *M. incognita* root population density statistically similar but numerically larger than the populations supported by the Rowden cultivar (**Table 3**). PhytoGen 800 is known to be highly resistant to FOV race 4; however, it was susceptible to all of the races and genotypes of FOV found in this study. Pima S-7 is a cultivar known to be susceptible to all FOV races. Deltapine 1558NR B2RF and PhytoGen 480 W3FE both have genes for resistance to *M. incognita* and sustained a significantly lower root population density of *M. incognita* compared to the Rowden susceptible check (**Table 3**). High levels of FOV infection were observed on these cultivars similar to that of the Rowden cultivar. However, M-315 and Stoneville 4946 GLB2 had low AUDPC values, 160 and 107 respectively. The AUDPC values for these cultivars were significantly reduced compared to the Rowden susceptible check and similar to that of the PhytoGen 72 cultivar (AUDPC 113), which is resistant to FOV race 1. M-315 is *M. incognita* resistant and sustained a lower nematode root population density compared to the Rowden susceptible check. Stoneville 4946 GLB2 supported a similar *M. incognita* root population density to Rowden control. However, this cultivar sustained less FOV damage than did than Rowen, as measured by AUDPC.

**Table 3:** *Meloidogyne incognita* race 3 average root population density by cotton cultivar from all *Fusarium* testing during 2018 and 2019 in the National Cotton Fusarium Wilt Evaluation field.

Cotton cultivar	*M. incognita* eggs/g of root^y^	*M. incognita* Reported resistance traits
Rowden	230^z^ cd	Susceptible
M-315	11 ab	Resistant
PHY 480 W3FE	4 a	Resistant
DeltaPine 1558NR B2RF	38 ab	Resistant
Stoneville 4946 GLB2	40 bc	Moderate Resistance
PhytoGen 72	125 cd	Susceptible
PhytoGen 800	1039 d	Susceptible
Pima S-7	790 d	Susceptible

y Meloidogyne incognita eggs were extracted from two cotton roots collected at the end of each growing season just before defoliation of the crop. Values present are means taken all three site years of the testing.

z Values present are LS-means of M. incognita root population density across all three tests, separated using Tukey’s HSD test at P ≤ 0.05. Nematode egg data were log-transformed to satisfy the ANOVA assumption of normally distributed residuals values in the column followed by different letters differ significantly.

### Temporal distribution of FOV

**Figure 7:**
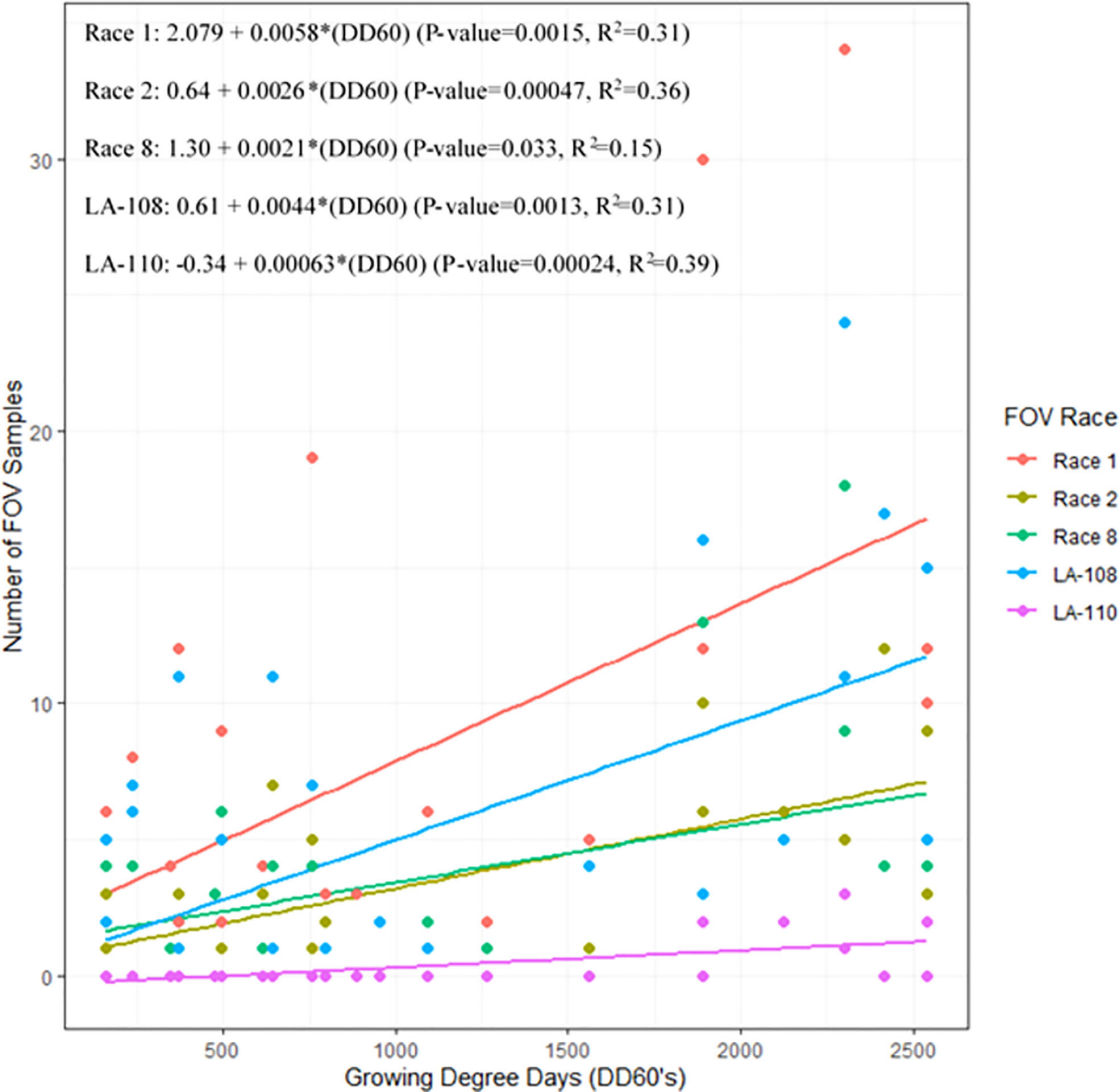
The 2018 and 2019 temporal distribution of *Fusarium oxysporum* f. sp. *vasinfectum* (FOV) throughout the cotton growing season. Graph shows the linear relationship between the number of FOV samples collected and the accumulation of growing degree days (DD60’s). Linear relationships for race 1 race 2, race 8, LA-108, and LA-110 are shown. Other races and genotypes found in this study did not have a significant relationship with accumulation of DD60’s and therefore are not shown in this figure.

Infection from certain races and genotypes of FOV was affected by DD60 accumulation (**Figure 7**). Race 1 FOV was found in every sampling date throughout the season, and the number of samples collected was correlated with the accumulation of DD60’s. FOV race 1 samples increased by 0.58 FOV samples/collection date ( ± 0.33; ± 95% C.I.) for every 100 DD60’s accumulated (P-value=0.0015, R^2^ = 0.31). Genotype LA-108 samples increased by 0.44 FOV samples/collection date (± 0.25; ± 95% C.I.) for every 100 DD60’s accumulated (P-value=0.0013, R^2^ = 0.31). Race 2 and 8 had similar rates of development to each other with a 0.26 FOV samples/collection date increase ( ± 0.13; ± 95% C.I.) for every 100 DD60’s accumulated (P-value=0.00047, R^2^ = 0.36), and a 0.21 FOV samples/collection date increase ( ± 0.19; ± 95% C.I.) in race 8 FOV samples for every 100 DD60’s accumulated (P-value=0.033, R^2^ = 0.15). Ten LA-110 samples were collected, and a significant correlation was observed between these samples and the accumulation of DD60’s. In LA-110 a 0.06 FOV samples/collection date increase ( ± 0.031; ± 95% C.I.) was observed for every 100 DD60’s accumulated (P-value=0.00024, R^2^ = 0.39). Other genotypes of FOV (LA-112, LA-127/140, and MDS-12) collected during this study were not found to have a significant correlation with DD60 accumulation. However, relatively low numbers (3, 13, and 16 isolates respectively) of these genotypes were found in this research.

## Discussion

The National Cotton *Fusarium* Wilt Evaluation Field was found to have a more diverse population of FOV races/genotypes than were found in previous studies by Smith (2015) and Scott (2012). In total, eight races/genotypes of FOV were found during our study. Smith (2015) did not detect the genotypes MDS-12 and LA-112 in this field. However, Scott (2012) did find the genotype MDS-12 but not LA-112. In our study, LA-112 was isolated at a very low frequency. Lack of detection of this genotype by Scott (2012) and Smith (2015) suggests the genotype was below detectable levels at the time of the studies. These results suggest that other fields may have more diverse FOV races/genotypes than previously recognized and such fields can only be characterized through an extensive sampling of symptomatic plants.

Another important result of this study is what was not found. FOV race 4 was not detected in the National Cotton *Fusarium* Wilt Evaluation Field, despite the many germplasm lines introduced and tested at this site for at least the last 60 years. To date, FOV race 4 has not been found in the state of Alabama and has only been reported in California (Kim et al., 2005), (Halpern et al., 2017), and New Mexico (Zhu et al., 2019c). In this testing some isolates were found to be identical to FOV race 4 and MDS-12 in the *EF-1*α, *BT*, and *PHO* gene sequences and sequencing IGS gene determined the identity of these isolates as MDS-12.

Race 4 is a highly virulent race of the FOV pathogen, which is not dependent on nematodes to cause high levels of damage. The most effective management strategy for this race of FOV is the use of tolerant cotton cultivars. To this point, there are Pima cotton cultivars with high levels of tolerance to FOV race 4 but so far, no Acala or Upland cultivars have been identified that have high levels of tolerance. In contrast, MDS-12 isolates collected in Alabama and Mississippi are of less of a concern because they were only moderately virulent to the cotton cultivars that have been tested (Bennett et al., 2013). Each of the 16 isolates, which shared the base sequence identity of the three diagnostic genes with FOV race 4, were confirmed to be MDS-12. This genotype has even been referred to as non-pathogenic (Bell et al., 2017). While the isolates collected in this study exhibited low virulence, they were capable of infecting cotton and causing disease on both Upland cotton (*G. hirsutum*) and Pima cotton (*G. barbadense*) under field conditions in the presence of *M. incognita*.

The FOV genotype of LA-112 was only found in 2019 which was a high disease incidence year over all compared to 2018. A low rate of infection and sparse distribution within the testing area suggests a recent introduction to the field. This isolate was not found during a survey of this field conducted during 2010 and 2011 (Scott, 2012) or 2013 and 2014 (Smith, 2015). However, the genotype LA-112 was found on this farm (Kathy Lawrence, Auburn University, personal communication) in samples submitted to a nationwide survey (Cianchetta et al., 2015). LA-112 may have been introduced into the area through contaminated seed produced in fields containing this genotype of FOV (Taubenhaus and Ezekiel, 1932). Greater distribution of this genotype can also be expected with continuous cotton production as it is spread within the field by cultivation or movement with water (Grinstein et al., 1983).

Seedborne dissemination likely played a role in the diversity of FOV race genotypes that were discovered in the studied field. This field has been monocultured in cotton for more than 60 years as the testing site of new cotton cultivars and breeding lines for resistance to *Fusarium* wilt). Each year a large number of new cotton cultivars and breeding lines from breeders around the world were screened in this field and the seeds could have brought new and different FOV races/genotypes as seedborne inoculum. Due to this continuous introduction of germplasm from diverse locations, we expect that this field evidences a more diverse FOV community than a typical farmers field, where one or two cotton cultivars may be planted each year. However, in this study, no Alabama farmer’s fields were surveyed for FOV diversity to confirm this. Georgia farmer’s fields have been reported to sometimes be infested with multiple races/genotypes of FOV (Bell et al., 2019). While this particular field may be unique in its history and abundant diversity of FOV, farmers could face similar problems with multiple populations of FOV causing damage to their cotton.

The most prevalent races/genotypes found in this study including race 1, race 2, race 8, and LA-108 accounted for more than 93% of the samples collected. All of these races/genotypes, along with LA-110, had a positive correlation with DD60 accumulation indicating that as the season progressed and cotton matured, higher rates of FOV symptoms were documented. This trend to later symptom expression partially mitigates damage. Plants which develop symptoms after flowering are often still capable of producing bolls though they may be reduced in number, size, and lint quality (Davis et al., 2006). In contrast, plants that are symptomatic early in the season often die and therefore produce no cotton. This trend of later disease expression is in contrast to studies with FOV race 4, a race not found in this testing, which has often been associated with early season (4-10 node stage) infections in California (Hutmacher et al., 2011).

The 2019 cotton season proved to be a severe FOV infection year compared to the 2018 season. Of the 661 total FOV samples collected during this study, 81% (535 samples) were collected during the 2019 testing versus only 126 collected in 2018. While there were twice as many plots (two tests) sampled in 2019, this increase in the number of plots fails to fully account for such a substantial increase in the number of samples. Environmental conditions, especially temperature, have been found to have an effect on the incidence and severity of FOV infection (Zhang et al., 2020). One major difference between the 2018 and 2019 seasons was the date of planting the cotton test. In 2018 the test was planted on May 17, and in 2019 the tests were planted on April 24. The earlier planting date resulted in cool and wet conditions during the early part of the season during 2019, unlike the 2018 test. During the first 30 days of the growing season in 2018, the cotton received 10.6 cm of rain, and 551 DD60’s had accumulated. During this same amount of time in 2019, the crop received nearly three centimeters more rain (13.0 cm), and only 350 DD60’s had accumulated. This difference in environmental conditions between the two seasons could have significantly affected the disease pressure throughout the year as has been observed with Fusarium wilt affecting many crops around the world (Scott et al., 2010; Li et al., 2017; Jelínek et al., 2019). Zhang et al. (2020) documented increased disease severity with some races of FOV with lower temperatures in the early part of the growing season which resulted in reduced DD60 accumulation.

Only one cultivar, PhytoGen 800, sustained more damage from FOV in 2018 compared to the 2019 season. This cultivar had an AUDPC rating of 305.5 in 2018 and 294.7 in 2019. This higher disease progress curve rating in what was generally the less severe of the two years may have been a result of higher nematode pressure on the variety in the 2018 cotton season. This cultivar had an average *M. incognita* root population density of 2,069 eggs/g of root during the 2018 season and 524 eggs/g of root during the 2019 season (data not shown). In both cases, this cultivar sustained the highest nematode root population density of any of the cultivars included in the test. It is unclear why the nematode population density was so high on this cultivar during the 2018 season. There is a strong interaction between *M. incognita* and FOV, this higher nematode population density could have increased the damage from the *Fusarium* wilt disease complex (Garber et al., 1979; DeVay et al., 1997).

Even though the disease complex of FOV and *M. incognita* has been recognized since the first reporting of *Fusarium* wilt, the specifics of this interaction are not fully understood. It is commonly thought that the increase in *Fusarium* wilt is the result of wounds created by nematode penetration and feeding. However, evidence suggests there is a more complex interaction due to physiological changes in the cotton root apart from wounds caused by the nematode (Starr, 1998). One of the driving forces behind this theory is the greenhouse observation that FOV infection is more severe when *M. incognita* is inoculated on the plants about four weeks before inoculation with FOV (Porter and Powell, 1967). *M. incognita* has also been found to cause little wounding to plant roots during the infection process suggesting that the interaction may rely on other mechanisms (Endo and Wergin, 1973). Giant cells, which are established for nematode feeding, are more susceptible to fungal infections, including infection by FOV which could play a role in the interaction of these two pathogens (Meléndez and Powell, 1967; Fattah and Webster, 1983). While the mechanism of this interaction may not be understood, the effects have been well documented (Atkinson, 1982; Roberts et al., 1985; Starr et al., 1989). The field used for this study has been infected with both the nematode and FOV pathogens for at least the last 60 years, and both pathogens continue to cause damage at this location.

Often the FOV-*M. incognita* disease complex is managed by reducing nematode population density in the cotton field (Jorgenson, 1978; Colyer et al., 1997). Whereas there are measures, such as rotations, nematicides, and resistant cultivars that are partially effective against *M. incognita*, there is a general lack of effective control methods against FOV (Hutmacher et al., 2011). Lowering nematode population density has been demonstrated to reduce the amount of FOV infection caused by vascular-competent races/genotypes (Colyer et al., 1997). While lowering the *M. incognita* population density in the field can reduce the damage from all races/genotypes of FOV found in the study field, this research demonstrated that plant resistance to the FOV as expressed in these cultivars appears to provide better protection than did *M. incognita* resistance. The cultivar PhytoGen 72, which is known to be widely resistant to FOV races/genotypes (Hutmacher et al., 2011), except for FOV race 4, had low levels of FOV infection even though it sustained a similar *M. incognita* root population density to that of the Rowden susceptible control. While the *M. incognita* resistant cultivars of Deltapine 1558NR B2RF and PhytoGen 480 W3FE did support a lower nematode population density, FOV disease incidence was not reduced. No significant correlation was observed between FOV race/genotype and *M. incognita* population density. FOV races/genotypes did not vary significantly in their distribution between the nematode resistant and nematode susceptible cultivars. This suggest that the FOV races/genotypes found in this study all interact synergistically with *M. incognit*a to cause *Fusarium* wilt and no isolates of the root-rot pathotypes are present at detectable levels in the field.

Other species of plant-parasitic nematodes have also been associated with an increased incidence of FOV, including *Rotylenchulus reniformis* and *Belonolaimus* (Jones et al., 1959; Khadr et al., 1972; Yang et al., 1975; Silva et al., 2019). *B. longicaudatus* has been associated with many fields incurring losses from *Fusarium* wilt in Georgia in the last few years and is speculated to play a larger role in the *Fusarium* wilt-nematode disease complex than previously thought (Silva et al., 2019). Neither *R. reniformis* nor *B. longicadutaus* were detected in the field location used in this study. This is most likely due to soil texture at the research location, which was a Kalmia loamy sand soil type consisting of 80% sand, 10% silt, and 10% clay. This soil texture is well suited for *M. incognita* but not for *R. reniformis* which is better suited to more finely textured silty soils or for *B. longicadutaus*, which require higher sand contents (Robbins and Barker, 1974; Robinson et al., 1987). The *Fusarium* wilt-nematode disease complex in this study is believed to rely entirely upon the interaction between FOV and *M. incognita*, as neither of the other two nematodes were found in the test field. A more in-depth understanding of the interactions of FOV races and genotypes and different plant-parasitic nematodes may help in the future management of this disease complex.

The *Fusarium* wilt-nematode disease complex of cotton is a multifaceted pathogenicity system made up of many races/genotypes of the FOV pathogen and numerous plant pathogenic nematodes. The apparent diversity of FOV is increasing with new genotypes of the pathogen being discovered and new species of *Fusarium* appearing to cause symptoms similar to *Fusarium* wilt (Guo et al., 2015; Zhu et al., 2019a; Zhu et al., 2019b). In China, a new genotype of FOV has been found to cause *Fusarium* wilt of cotton. These newly described genotypes are distinctly different from previously described genotypes and races FOV in China (Guo et al., 2015). Additional *Fusarium* species (e.g. *F. proliferatum* and *F. solani*) have been also identified to engender wilt of cotton in New Mexico, producing symptoms of leaf yellowing and wilting as well as discolored vascular tissue (Zhu et al., 2019a; Zhu et al., 2019b). With the genetic variation that exists within this disease complex, more in-depth studies are needed to understand the relationships between these races/genotypes and how the disease may be managed. Recent work in full genome sequencing (Seo et al., 2020) may help to provide some insight into the relationships of this diverse group of cotton pathogens. With the spread of highly virulent races of the pathogen such as FOV race 4, better understanding of the disease interactions and options for management are of increasing importance.

## Data availability statement

The datasets presented in this study can be found in online at https://www.ncbi.nlm.nih.gov/genbank/, with accession numbers: ON409993-ON411130.

## Author contributions

DD and KL designed the nematode experiments. DD and KL performed the experiments and the data collection. DD ran all the PRC and fungal identification. MN deposited the sequences in Genebank. DD and KL drafted the manuscript. DD and KL wrote the final version of the manuscript. All authors edited and approved the final manuscript.

## Funding

This research was partially funded by Cotton Inc. Cotton Inc 16-241AL and Hatch project ALA15-214003.

## Acknowledgments

Authors would like to thank Dr. Robert Nichols for his assistance in the project design and review of this manuscript and Dr. Patricia Donald for her through editing of the manuscript.

## Conflict of interest

The authors declare that this study received funding from Cotton Inc. The funder was involved in cotton varieties selected and not involved in the study design, collection, analysis, interpretation of data, the writing of this article, or the decision to submit it for publication.

## Publisher’s note

All claims expressed in this article are solely those of the authors and do not necessarily represent those of their affiliated organizations, or those of the publisher, the editors and the reviewers. Any product that may be evaluated in this article, or claim that may be made by its manufacturer, is not guaranteed or endorsed by the publisher.

## References

[B1] Armstrong J. K. Armstrong G. M. (1958). A race of the cotton-wilt *Fusarium* causing wilt on yelredo soybean and flue-cured tobacco. Plant Dis. Rep. 42, 147–151.

[B2] Atkinson G. F. (1982). Some diseases of cotton. Agric. Experiment. Station Bull. Agric. Mechanical. Coll. 41, 1–65.

[B3] Bell A. A. Gu A. Olvey J. Wanger T. A. Tashpulatov J. J. Prom S. . (2019). Detection and characterization of fusarium oxysporum f. sp. *vasinfectum* VCG0114 (Race 4) isolates of diverse geographic origins. Plant Dis. 103, 1998–2009. doi: 10.1094/PDIS-09-18-1624-RE 31188737

[B4] Bell A. A. Kemerait R. C. Ortiz C. S. Prom S. Quintana J. Nichols R. L. . (2017). Genetic diversity, virulence, and *Meloidogyne incognita* interactions of *Fusarium oxysporum* isolates causing wilt in Georgia. Plant Dis. 101, 948–956. doi: 10.1094/PDIS-09-16-1382-RE 30682930

[B5] Bennett R. S. Scott T. Z. Lawrence K. S. Lawrence, G. W. (2013). Sequence characterization of race 4-like isolates of *Fusarium oxysporum* from Alabama and Mississippi. J. Cotton. Sci. 17, 125–130.

[B6] Cianchetta A. N. Allen T. W. Hutmacher R. B. Kemerait R. C. Kirkpatrick T. L. Lawrence G. W. . (2015). Survey of *Fusarium oxysporum* f. sp. *vasinfectum* in the united states. J. Cotton. Sci. 19, 328–336.

[B7] Colyer P. D. Kirkpatrick T. L. Caldwell W. D. Vernon, P. R. (1997). Influence of nematicide application on the severity of root-knot nematode-fusarium wilt disease complex in cotton. Plant Dis. 81, 66–70. doi: 10.1094/PDIS.1997.81.1.66 30870950

[B8] Crop Protection Network (2022). Estimates of crop yield losses due to diseases and invertebrate pests: an online tool. doi: 10.31274/cpn-20191121-0

[B9] Davis R. D. Colyer P. D. Rothrock C. S. Kochman J. K. (2006). Fusarium wilt of cotton: Population diversity and implication for management. Plant Dis. 90, 692–703. doi: 10.1094/PD-90-0692 30781226

[B10] Davis R. D. Moore N. Y. Kochman J. K. (1996). Characterization of a population of *Fusarium oxysporum* f. sp. *vasinfectum* causing wilt of cotton in Australia. Aust. J. Agric. Res. 47, 1143–1156. doi: 10.1071/AR9961143

[B11] DeVay J. E. Gutierrez A. P. Wakeman R. J. Garber R. H. Jeffers D. P. . (1997). Inoculum densities of *Fusarium oxysporum* f. sp. *vasinfectum* and *Meloidogyne incognita* in relation to the development of fusarium wilt and the phenology of cotton plants (*Gossypium hirsutum*). Phytopathol. 87, 341–346. doi: 10.1094/PHYTO.1997.87.3.341 18945178

[B12] Doan H. K. Davis R. M. (2014). Evaluation of fusarium wilt resistance in six upland cotton germplasm lines. J. Cotton. Sci. 18, 430–434.

[B13] Edel-Hermann V. Lecomte C. (2019). Current status of *Fusarium oxysporum* formae speciales and races. Phytopathol. 109, 512–530. doi: 10.1094/PHYTO-08-18-0320-RVW 30461350

[B14] Endo B. Y. Wergin W. P. (1973). Ultrastructural investigation of clover roots during early stages of infection by the root-knot nematode *Meloidogyne incognita* . Protoplasma 78, 365–379. doi: 10.1007/BF01275773

[B15] Faske T. R. Overstreet C. Lawrence G. Kirkpatrick T. L. (2018). “Important plant parasitic nematodes of row crops in Arkansas, Louisiana, and mississippi” in plant parasitic nematodes in sustainable agriculture of north America Vol. Vol. 2. Eds. Subbotin A. A. Chitambar J. J. (New York, NY: Springer), 393–432. Northeastern, Midwestern, and Southern USA.

[B16] Fattah F. Webster J. M. (1983). Ultrastructural changes caused by *Fusarium oxysporum* f sp. *lycopersici* in *Meloidogyne javanica* induced giant cells in *Fusarium* resistant and susceptible tomato cultivars. J. Nematol. 15, 128–135. 19295778 PMC2618247

[B17] Fernandez D. Assigbetse K. Dubois M. Geiger J. (1994). Molecular characterization of races and vegetative compatibility groups in *Fusarium oxysporum* f. sp. *vasinfectum* . Appl. Environ. Microbiol. 60, 4039–4046. doi: 10.1128/aem.60.11.4039-4046.1994 7993090 PMC201933

[B18] Garber R. H. Jorgenson E. C. Smith S. Hyer A. H. (1979). Interaction of population levels of *Fusarium oxysporum* f. sp. *vasinfectum* and *Meloidogyne incognita* on cotto. J. Nematol. 11, 133–137. 19305546 PMC2617958

[B19] Grinstein A. Fishler G. Katan J. Hakohen D. (1983). Dispersal of the fusarium wilt pathogen in furrow-irrigated cotton in Israel. Plant Dis. 67, 742–743. doi: 10.1094/PD-67-742

[B20] Groover W. Lawrence K. S. Donald P. (2020). Reproductive rate differences of root-knot nematode from multiple crops in a single field. Nematropica 49, 152–156.

[B21] Guo Q. Li S. Lu X. Gao H. Wang X. Ma Y. . (2015). Identification of new genotypes of *Fusarium oxysporum* f. sp. *vasinfectum* on cotton in China. Plant Dis. 99, 1569–1577. doi: 10.1094/PDIS-12-14-1238-RE 30695955

[B22] Hall T. A. (1999). BioEdit: a user-friendly biological sequence alignment editor and analysis program for windows 95/98/NT. Nucleic Acids Symp. Ser. 41, 95–98.

[B23] Halpern H. C. Bell A. A. Wagner T. A. Liu J. Nichols R. L. Olvey J. . (2017). First report of fusarium wilt of cotton caused by *Fusarium oxysporum* f. sp. *vasinfectum* race 4 in Texas, USA. Plant Dis. 102, 446–446. doi: 10.1094/PDIS-07-17-1084-PDN

[B24] Holmes E. A. Bennett R. S. Spurgeon D. W. Colyer P. D. Davis R.M. (2009). New genotypes of *Fusarium oxysporum* f. sp. *vasinfectum* from the southeastern united states. Plant Dis. 93, 1298–1304. doi: 10.1094/PDIS-93-12-1298 30759505

[B25] Hussey R. S. Barker K. B. (1973). A comparison of methods of collecting inocula for meloidogyne spp., including a new technique. Plant Dis. Rep. 57, 1025–1028.

[B26] Hutmacher R. B. Ulloa M. Wright S. D. Campbell B. T. Percy R. Wallace T. . (2013). Elite upland cotton germplasm-pool assessment for fusarium wilt resistance in California. Agron. J. 105, 1635–1644. doi: 10.2134/agronj2013.0264

[B27] Hutmacher R. B. Ulloa M. Wright S. D. Davis R. M. Kelly M. P. Delgado R. . (2011). “Fusarium race 4: management recommendations for growers”,” in Proceedings of the beltwide cotton conference. Eds. Boyd S. Huffman M. Robertson B. (Cordova, TN: NCCA), 188–192.

[B28] Jelínek T. Koudela M. Kofránková V. Salava J. (2019). Effect of temperature on severity of fusarium wilt of cabbage caused by *Fusarium oxysporum* f. sp. *conglutianans* . Eur. J. Plant Pathol. 155, 1277–1286. doi: 10.1007/s10658-019-01855-3

[B29] Jenkins W. R. (1964). A rapid centrifugal-flotation technique for separating nematodes from soil. Plant Dis. Rep. 48, 692.

[B30] Jorgenson E. C. (1978). Effects of aldicarb on fusarium wilt-root-knot nematode disease of cotton. J. Nematol. 10, 372–374. 19305871 PMC2617913

[B31] Jones J. E. Newsom L. D. Finley E. L. (1959). Effect of the reniform nematode on yield, plant characters, and fiber properties of upland cotton. Agron. J. 51, 353–356.

[B32] Khadr A. S. Salem A. A. Oteifa B. A. (1972). Varietal susceptibility and significance of the reniform nematode *Rotylenchulus reniformis*, in fusarium wilt of cotton. Plant Dis. Rep. 56, 1040–1042.

[B33] Kim Y. Hutmacher R. B. Davis R. M. (2005). Characterization of California isolates of *Fusarium oxysporum* f. sp. *vasinfectum* . Plant Dis. 89, 366–372. doi: 10.1094/PD-89-0366 30795451

[B34] Kumar S. Stecher G. Li M. Knyaz C. Tamura K. (2018). MEGAX: Molecular evolutionary genetics across computing platforms. Mol. Biol. Evol. 35, 1547–1549. doi: 10.1093/molbev/msy096 29722887 PMC5967553

[B35] Liu J. Bell A. A. Wheeler M. H. Stipanovic R. D. Puckhaber L. S. (2011). Phylogeny and pathogenicity of *Fusarium oxysporum* isolates from cottonseed imported from Australia into California for dairy cattle feed. Can. J. Microbiol. 57, 874:886. 22004096 10.1139/w11-080

[B36] Li X. Zhang Y. Ding C. Xu W. Wang X. (2017). Temporal patterns of cotton fusarium and verticillium wilt in jiangsu coastal areas of China. Sci. Rep. 7, 12581. doi: 10.1038/s41598-017-12985-1 28974768 PMC5626778

[B37] Mahill J. F. Pellow J. W. (2005). Cotton cultivar PHY 800 pima (U.S. Patent Application Publication). Publication number US205/0138683A1.

[B38] Meléndez P. L. Powell N. T. (1967). Histological aspects of the fusarium wilt-root-knot complex in flue-cured tobacco. Phytopathol. 57, 286–292.

[B39] Mendiburu F. (2020)Agricolae: statistical procedures for agricultural research. In: R package version 1.3-2. Available at: https://CRAN.R-project.org/package=agricolae (Accessed August 18, 2022).

[B40] Nelson P. E. Toussoun T. A. Marasas W. F. O. (1983). “Section elegans,” in Fusarium species: An illustrated manual for identification, 142–145. The Pennsylvania State University Press University Park, Pennsylvania.

[B41] O’Donnell K. Gueidan C. Sink S. Johnston P. R. Crous P. W. Glenn A. . (2009). A two-locus DNA sequence database for typing plant and human pathogens within the fusarium oxysporum species complex. Fungal Genet. Biol. 46, 936–948. doi: 10.1016/j.fgb.2009.08.006 19715767

[B42] O’Donnell K. Kistler H. C. Cigelnik E. Ploetz R. C. (1998). Multiple evolutionary origins of the fungus causing Panama disease of banana: concordant evidence from nuclear and mitochondrial gene genealogies. Proc. Natl. Acad. Sci. U.S.A. 95, 2044–2049. doi: 10.1073/pnas.95.5.2044 9482835 PMC19243

[B43] O’Donnell K. Kistler H. C. Tacke B. K. Casper H. H. (2000). Gene genealogies reveal global phylogeographic structure and reproductive isolation among lineages of *Fusarium graminearum*, the fungus causing wheat scab. Proc. Natl. Acad. Sci. U.S.A. 97, 7905–7910. doi: 10.1073/pnas.130193297 10869425 PMC16643

[B44] Ortiz C. S. Bell A. A. Magill C. W. Liu J. (2017). Specific PCR detection of *Fusarium oxysporum* f. sp. *vasinfectum* California race 4 based on a unique Tƒo1 insertion event in the PHO gene. Plant Dis. 101, 34–44. doi: 10.1094/PDIS-03-16-0332-RE 30682321

[B45] Porter D. M. Powell N. T. (1967). Influence of certain *Meloidogyne* species on fusarium wilt development in flue-cured tobacco. Phytopathol. 57, 282–285.

[B46] R Core Team (2018) R: A language and environment for statistical computing. Available at: http://www.r-prodject.org/ (Accessed August 18, 2022).

[B47] Robbins R. T. Barker K. R. (1974). The effects of soil type, particle size, temperature, and moisture, on reproduction of *Belonolaimus longicaudatus* . J. Nematol. 6, 1–6. 19319355 PMC2620035

[B48] Roberts P. A. Smith S. N. Matthews W. C. (1985). Quantitative aspects of the interaction of Meloidogyne incognita with Fusarium wilt on Acala cotton in field plots. Pages 21-22 in *Proceedings of the Beltwide Cotton Conference* . Dugger C. Richter D. , eds. National Cotton Council of America: Cordova, TN.

[B49] Robinson A. F. Heald C. M. Flanagan S. L. Thames W. H. Amador J. (1987). Geographical distribution of *Rotylenchulus reniformis*, *Meloidogyne incognita*, and *Tylenchulus semipenetrans* in the lower Rio grande valley as related to soil texture and land use. Ann. Appl. Nematol. 1, 20–25. PMC2618697 19290268

[B50] RStudio Team (2015) RStudio: integrated development for r. RStudio (Boston, MA). Available at: http://www.rstudio.com/ (Accessed August 18, 2022).

[B51] Schandry N. (2017). A practical guide to visualization and statistical analysis of r. solanacearum infection data using r. front. Plant Sci. 8. doi: 10.3389/fpls.2017.00623 PMC5401893 28484483

[B52] Scott J.C. Gordon T. R. Shaw D. V. koike S. T. (2010) Effect of temperature on severity of fusarium wilt of lettuce caused by *Fusarium oxysporum* f. sp. *lactucae* Plant Dis. 94, 13–17. doi: 10.1094/PDIS-94-1-0013 30754388

[B53] Scott T. Z. (2012). Cultivar susceptibility of fusarium wilt complex and race characterization of fusarium oxysporum f. sp. vasinfectum (Auburn, AL.: Auburn University). Available at: http://hdl.handle.net/10415/3120.

[B54] Seo S. Pokhrel A. Coleman J. (2020). The genome sequence of five genotypes of *Fusarium oxysporum* f. sp. *vasinfectum*: a resource for studies on fusarium wilt of cotton. Molecular Plant-Microbe Interactions 33, 138–140. doi: 10.1094/MPMI-07-19-0197-A 31593526

[B55] Silva M. B. Davis R. F. Doan H. K. Nichols R. L. Kemerait R. C. Halpern H. C. . (2019). Fusarium wilt of cotton may commonly result from the interaction of *Fusarium oxysporum* f. sp. *vasinfectum* with *Belonolaimus longicaudatus* . J. Nematol. 51, 1–10. doi: 10.21307/jofnem-2019-015 PMC6929639 31088027

[B56] Smith A. L. (2015). Identification of resistant or tolerant commercial cotton cultivars to the fusarium wilt root-knot nematode disease complex and the identification fusarium oxysporum f. sp. vasinfectum races in Alabama (Auburn (AL: Auburn University). Available at: http://hdl.handle.net/10415/4563.

[B57] Starr J. L. (1998). “Cotton”, in Plant nematode interactions. Eds. Barker K. R. Pederson G. A. Windham G. L. (Madison, WI: Amer. Soc. Agro), 359–379.

[B58] Starr J. L. Jeger M. J. Martyn R. D. Schilling K. (1989). Effects of meloidogyne incognita and fusarium oxysporum f. sp. vasinfectum on plant mortality and yield of cotton,Phytopathology 79. 640–646.

[B59] Shepherd R. L. McCarty J. C. Jenkins J. N. Parrott W. L. (1996). Registration of nine cotton germplasm lines resistant to root-knot nematode. Crop Sci. 36, 820. doi: 10.2135/cropsci1996.0011183X003600030071x

[B60] Tamura K. Nei M. (1993). Estimation of the number of nucleotide substitutions in the control region of mitochondrial DNA in humans and chimpanzees. Mol. Biol. Evol. 10, 512–526. 8336541 10.1093/oxfordjournals.molbev.a040023

[B61] Taubenhaus J. J. Ezekiel W. N. (1932). Seed transmission of cotton wilt. Science 76, 61–62. doi: 10.1126/science.76.1959.61 17847304

[B62] Tooley P. W. Goley E. D. Carras M. M. Frederick R. D. Weber E. L. Kuldau G. A. (2001). Characterization of *Clavicpes* species pathogenic on sorghum by sequence analysis of the b-tubulin gene intron 3 region and EF-1a gene intron 4. Mycologia 93, 541–551. doi: 10.1080/00275514.2001.12063186

[B63] Turcotte E. L. Percy R. G. Feaster C. V. (1992). Registration of ‘Pima s-7’ American pima cotton. Crop Sci. 32, 1291. doi: 10.2135/cropsci1992.0011183X003200050047x

[B64] Wickham H. (2016). Ggplot2: elegant graphics for data analysis (New York, NY: Springer Verlag).

[B65] Yang M. E. Davis R. M. Hutmacher R. B. (2006). “Fusarium wilt of cotton in California: characterization and PCR-based detection of race 4” in Proceedings of the beltwide cotton conference. Eds. Boyd S. Huffman M. Robertson B. (Cordova, TN: NCCA), 93–96.

[B66] Yang H. Powell N. T. Barker K. R. (1975). Interaction of concomitant species of nematodes and *Fusarium oxysporum* f. sp. *vasinfectum* on cotton. J. Nematol. 8, 74–80. PMC2620153 19308201

[B67] Zhang J. Abdelraheem A. Zhu Y. Wheeler T. A. Dever J. K. Frelichowski J. . (2020). Assessing genetic variation for fusarium wilt race 4 resistance in tetraploid cotton by screening over three thousand germplasm lines under greenhouse or controlled conditions. Euphytica 216, 108. doi: 10.1007/s10681-020-02646-2

[B68] Zhu Y. Abdelraheem A. Sanogo S. Wedegaertner T. Nichols R. Zhang J. F. (2019a). First report of cotton (*Gossypium*) wilt caused by fusarium proliferatum in new Mexico. U.S.A. Plant Dis 103, 2679. doi: 10.1094/PDIS-04-19-0713-PDN

[B69] Zhu Y. Abdelraheem A. Sanogo S. Wedegaertner T. Nichols R. Zhang J. F. (2019b). First report of fusarium solani causing fusarium wilt in pima cotton (*Gossypium barbadense*) in new Mexico, USA. Plant Dis 103, 3279. doi: 10.1094/PDIS-05-19-1081-PDN

[B70] Zhu Y. Lujan P. A. Wedegaertner T. Nichols R. Abdelraheem A. Zhang J. F. . (2019c). First report of *Fusarium oxysporum* f. sp. *vasinfectum* race 4 causing fusarium wilt of cotton in new Mexico. Plant Dis 103:588. doi: 10.1094/PDIS-06-19-1170-PDNTABLE1 33543991

