# Supplementary material for: Diversity and temporal distribution of Fusarium oxysporum f. sp. vasinfectum races and genotypes as influenced by Gossypium cultivar
Source: Front Fungal Biol. 2022 Oct 20;3:1022761. doi: 10.3389/ffunb.2022.1022761 (PMC10512365; doi:10.3389/ffunb.2022.1022761)
Supplement: Supplementary file 1 [file DataSheet_1.zip › sequience data/FOV Trial Map.pdf]

Auburn University (Graduate Student)

In-Season Periodicity of Persistence and Infection of Fusarium oxysporum f. sp. vasinfectum (FOV) Genotypes in Cotton Prodject: 18-438

Trial ID: FOV 2018
Location: PBU Trial Year: 2018
Protocol ID: FOV Genotypes
Investigator: Kathy Lawrence
Project ID:
Study Director: David Dyer
Sponsor Contact: Bob Nichols

Trial Map Treatment Description

Trt	Code	Description
1		Rowden
2		M-315
3		ST 4946 GLB2
4		PHY 480 W3FE
5		DP 1558NR B2RF
6		Pima S7
7		PHY 800
8		PHY 72
9		Rowden
10		M-315

1001<br>7	1002<br>8	1003<br>1	1004<br>6	1005<br>9	1006<br>10	1007<br>5	1008<br>2	1009<br>4	1010<br>3
901<br>1	902<br>2	903<br>5	904<br>10	905<br>3	906<br>4	907<br>9	908<br>6	909<br>8	910<br>7
801<br>2	802<br>3	803<br>6	804<br>1	805<br>4	806<br>5	807<br>10	808<br>7	809<br>9	810<br>8
701<br>3	702<br>4	703<br>7	704<br>2	705<br>5	706<br>6	707<br>1	708<br>8	709<br>10	710<br>9
601<br>8	602<br>9	603<br>2	604<br>7	605<br>10	606<br>1	607<br>6	608<br>3	609<br>5	610<br>4
501<br>5	502<br>6	503<br>9	504<br>4	505<br>7	506<br>8	507<br>3	508<br>10	509<br>2	510<br>1
401<br>10	402<br>1	403<br>4	404<br>9	405<br>2	406<br>3	407<br>8	408<br>5	409<br>7	410<br>6
301<br>9	302<br>10	303<br>3	304<br>8	305<br>1	306<br>2	307<br>7	308<br>4	309<br>6	310<br>5
201<br>4	202<br>5	203<br>8	204<br>3	205<br>6	206<br>7	207<br>2	208<br>9	209<br>1	210<br>10
101<br>6	102<br>7	103<br>10	104<br>5	105<br>8	106<br>9	107<br>4	108<br>1	109<br>3	110<br>2
